# Supplementary material for: Candida albicans infection suppresses lipopolysaccharide or Pseudomonas aeruginosa stimulated murine bone marrow derived macrophage (BMDM) responses
Source: Sci Rep. 2026 Feb 13;16:8751. doi: 10.1038/s41598-026-39429-z (PMC12982582; doi:10.1038/s41598-026-39429-z)
Supplement: Supplementary file 2 — Supplementary Material 2 [file 41598_2026_39429_MOESM2_ESM.pdf]

Candida albicans infection suppresses Lipopolysaccharide or *Pseudomonas aeruginosa* stimulated murine bone marrow derived macrophage (BMDM) responses

Christa P. Baker[1], Stephanie Laba[1], Jordan Warner[1], Karen Shepherd[1], Heather M. Wilson[2], and J. Simon C. Arthur[1*]
1 Division of Cell Signalling and Immunology, School of Life Sciences, University of Dundee, Dow St. Dundee, DD15EH, UK.
2 Institute of Medical Sciences, University of Aberdeen, Foresterhill, Aberdeen, AB25 2ZD, UK

Supplementary Tables 2,4,6 and 8 (Supplementary Tables 1,3,5 are provided as separate excel sheets)

Supplementary Figures 1 – 9

Supplementary Table 2   Statistical testing for Figure 3

Figure	test		F	p	multiple comparisons test
3A	Two-way ANOVA	Interaction	81.46	*p*<0.0001	Šídák's
		+/- Infection	1132.5	*p* <0.0001	
		TLR agonist	108.7	*p* <0.0001	
3B	Welch's ANOVA		393	*p* <0.0001	Dunnett's T3
3C	Welch's ANOVA		85.69	*p* <0.0001	Dunnett's T3
3D		Interaction	273.0	*p* <0.0001	Šídák's
		+/- anti-IL-10	378.0	*p* <0.0001	
		stimulation	652.5	*p* <0.0001	
3E	Welch's ANOVA		379.6		Dunnett's T3
3F	Two-way ANOVA	Interaction	15.57	*p* =0.00005	Šídák's
		+/- insert	17.40	*p* =0.0007	
		stimulation	84.15	*p* <0.0001	
3G	Two-way ANOVA	Interaction	17.61	*p* =0.0003	Šídák's
		+/- LPS	372.5	*p* <0.0001	
		infection	17.55	*p* =0.0003	

Supplementary Table 4 Statistical testing for Figure 6, 8 and Supplemental Figure 7

Protein	Figure	test	ANOVA F	ANOVA p	multiple comparison test	Figure	test	ANOVA F	ANOVA p	multiple comparison test	Figure	test	ANOVA F	ANOVA p	multiple comparison test
IL-12p40	6A	Student's ttest			n.a.	SF6A	Student's ttest			n.a.	8A	Student's ttest			n.a.
TNF	6B	Student's ttest			n.a.	SF6B	one-way ANOVA	69.74	<0.0001	Dunnett's	8B	one-way ANOVA	44.2	<0.0001	Dunnett's
IL-1B	6C	Student's ttest			n.a.	SF6C	Student's ttest			n.a.	8C	Student's ttest			n.a.
IL-18	6D	Welch's ANOVA	8.938	0.0166	Dunnett's T3	SF6D	Welch's ANOVA			Dunnett's T3	8D	Welch's ANOVA	22.63	0.002	Dunnett's T3
IL-27	6E	Student's ttest			n.a.	SF6E	Student's ttest			n.a.	8E	Student's ttest			n.a.
IL-1RN	6F	one-way ANOVA	226.2	<0.0001	Dunnett's	SF6F	one-way ANOVA	31.15	<0.0001	Dunnett's	8F	one-way ANOVA	129	<0.0001	Dunnett's
CCL2	6G	one-way ANOVA	21.83	0.0003	Dunnett's	SF6G	Welch's ANOVA	48.29	0.0004	Dunnett's T3	8G	one-way ANOVA	13.39	0.0028	Dunnett's
CCL3	6H	Student's ttest			n.a.	SF6H	Student's ttest			n.a.	8H	Student's ttest			n.a.
CCL4	6I	Student's ttest			n.a.	SF6I	Student's ttest			n.a.	8I	Student's ttest			n.a.
CCL5	6J	Student's ttest			n.a.	SF6J	Student's ttest			n.a.	8J	Student's ttest			n.a.
CCL7	6K	Student's ttest			n.a.	SF6K	one-way ANOVA	39.76	<0.0001	Dunnett's	8K	Welch's ANOVA	57.43	0.0003	Dunnett's T3
CXCL2	6L	one-way ANOVA	24.7	0.0007	Dunnett's	SF6L	Welch's ANOVA	13.44	0.01	Dunnett's T3	8L	one-way ANOVA	37.76	0.0004	Dunnett's
CXCL10	6M	Welch's ANOVA	255.3	<0.0001	Dunnett's T3	SF6M	one-way ANOVA	90.97	<0.0001	Dunnett's	8M	one-way ANOVA	55.98	<0.0001	Dunnett's
CXCL16	6N	one-way ANOVA	10.86	0.0017	Dunnett's	SF6N	one-way ANOVA	17.14	0.002	Dunnett's	8N	one-way ANOVA	2.365	0.1323	Dunnett's
PTGS2	6O	one-way ANOVA	145.4	<0.0001	Dunnett's	SF6O	Student's ttest			n.a.	8O	one-way ANOVA	71.06	<0.0001	Dunnett's
NOS2	6P	Student's ttest			n.a.	SF6P	Student's ttest			n.a.	8P	Student's ttest			n.a.
ACOD1	6Q	Welch's ANOVA	2746	<0.0001	Dunnett's T3	SF6Q	one-way ANOVA	295.3	<0.0001	Dunnett's	8Q	one-way ANOVA	312.6	<0.0001	Dunnett's
ATF3	6R	one-way ANOVA	58.9	<0.0001	Dunnett's	SF6R	Welch's ANOVA	193	<0.0001	Dunnett's T3	8R	one-way ANOVA	44.83	<0.0001	Dunnett's
JUNB	6S	one-way ANOVA	247.3	<0.0001	Dunnett's	SF6S	Welch's ANOVA	137.5	<0.0001	Dunnett's T3	8S	one-way ANOVA	194.6	<0.0001	Dunnett's
CEBPB	6T	one-way ANOVA	84.36	<0.0001	Dunnett's	SF6T	Welch's ANOVA	85.26	<0.0001	Dunnett's T3	8T	Welch's ANOVA	207.3	<0.0001	Dunnett's T3

Supplementary Table 6 Primary antibody supplier list

Antibody	clone	Company	Cat. Number	Species	Dilution
GAPDH	14C10	Cell Signalling	2118s	Rabbit	1:1000
total p44/42 MAPK		Cell Signalling	9102L	Rabbit	1:1000
phospho-T202/Y204 p44/42 MAPK		Cell Signalling	9101L	Rabbit	1:1000
total p38, MAPK		Cell Signalling	9212L	Rabbit	1:1000
phospho-T180/Y182 p38 MAPK	D3F9 XP	Cell Signalling	4511L	Rabbit	1:1000
total- NF-κB p65	D14E12	Cell Signalling	8242s	Rabbit	1:1000
phospho-Ser536 NF-κB p65	94H1	Cell Signalling	3033s	Rabbit	1:1000
total CREB	48H2	Cell Signalling	9197L	Rabbit	1:1000
phospho-Ser133 CREB	87G3	Cell Signalling	9198L	Rabbi	1:1000
phospho-Ser933 NF-kappaB p105		Cell Signalling	4806s	Rabbit	1:1000
phospho-Ser172 TBK1	D52C2	Cell Signalling	5483s	Rabbit	1:1000
phospho-Thr183/Tyr185 JNK	81E11	Invitrogen	MA5-14943	Rabbit	1:1000

Supplementary Table 8 Statistical testing for Supplemental Figure 3

	Stimulation		Time		interaction	
Panel	F	p	F	p	F	p
A	1.946	0.1853	4.510	0.0552	1.798	0.2074
B	2.924	0.0924	2.067	0.1761	4.169	0.0422
C	2.043	0.1724	0.8622	0.3714	0.03022	0.9703
D	1.833	0.2098	0.5974	0.4574	0.2700	0.7687
E	516.9	P<0.0001	304.3	P<0.0001	130.9	P<0.0001
F	160.7	P<0.0001	142.0	P<0.0001	44.13	P<0.0001

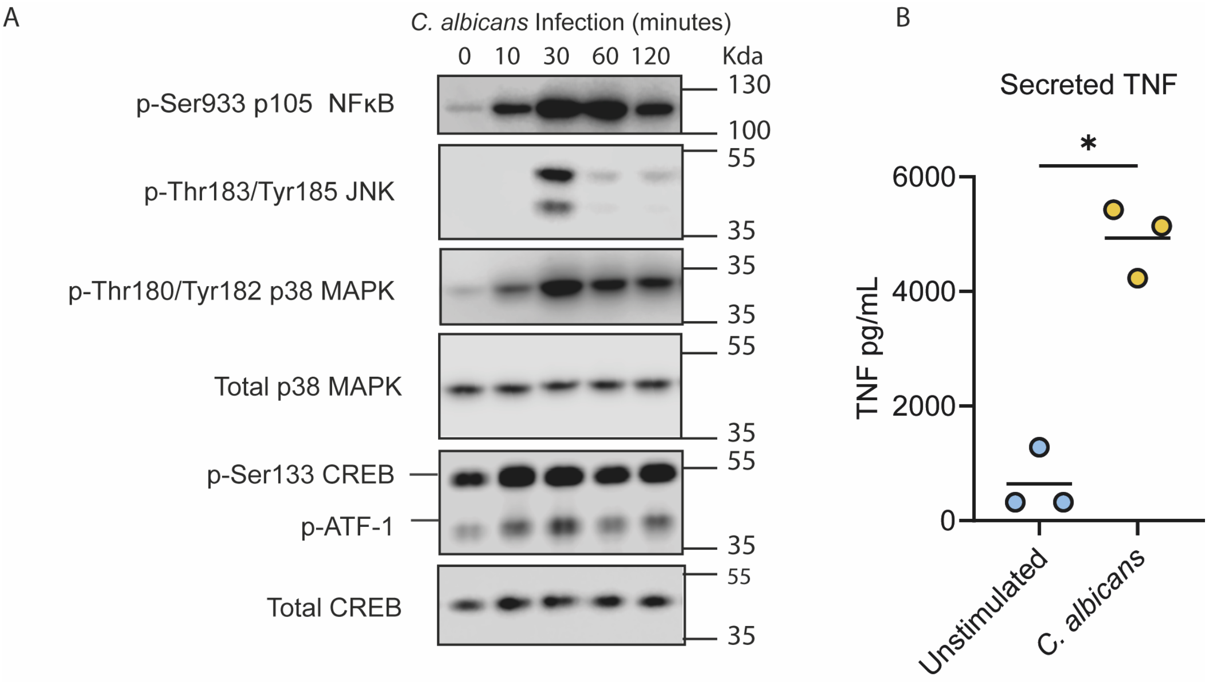

Supplementary Figure 1 ***C. albicans*** **infection can activate signalling and TNF production in BMDMs.** (A) BMDMs were left uninfected or infected with *C. albicans* with a multiplicity of infection (MOI) of 4 for the indicated times and lysed for immunoblot analysis to look at phospho-p105, phospho-JNK, phospho-p38, phospho-CREB/ATF1, total p38 and total CREB. (B) BMDMs were infected with *C. albicans* (MOI4) for 8 hours and then media was collected to analyse TNF levels. Graph shows results from 3 biological replicates with the mean value indicated by a line. Significance was determined by a two tailed Student's ttest, $p < 0.05$ is shown by *. Full images of blots are shown in supplementary figure 10.

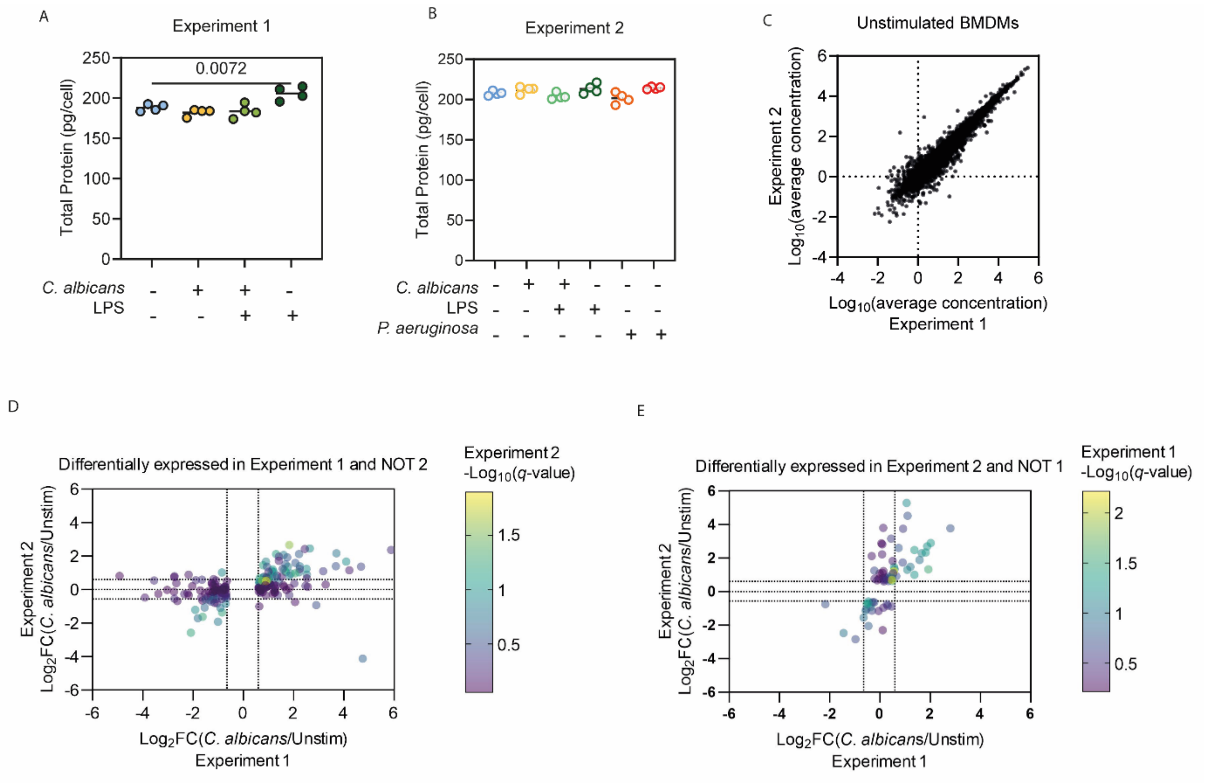

Supplementary Figure 2 Comparison of Proteomic experiments.

Two proteomic experiments were performed for this study. In the first. Macrophages from WT C57Bl/6 mice were either left unstimulated, infected with *C. albicans* at an MOI of 4, stimulated with100ng/ml of LPS or treated with both *C. albicans* and LPS, all for 8h. In the 2nd experiment macrophages from Ptpn1$^{fl/fl}$ LysM-Cre^{-ve} mice (wild type) were used. Ptpn1$^{fl/fl}$ LysM-Cre^{+ve} mice were also used, but showed minimal differences to the wild type cells and have therefore not been included in this report. Conditions in this 2nd study were the same as the 1st experiment but with 2 extra conditions, infection with *P. aeruginosa* and co-infection with both *P. aeruginosa* and *C. albicans*. 4 biological replicates were analysed in each experiment. (A, B) For proteomic experiments 1 and 2, the total protein per cell was estimated from the mass spectrometry using the histone Proteomic Ruler method as described in the methods. Data was analysed by one-way ANOVA and Tukey's multiple comparison testing. For experiment 1, F=10.56 and p=0.0011 while for experiment 2 F= 4.992 and p=0.0048. For comparisons to the unstimulated cells, p values of less than 0.05 are indicated on the graph. (C) Average protein concentrations were calculated for the unstimulated BMDM replicates within each experiment. Graph show the log10 of the average concentrations for proteins identified in the proteomics in both experiments. The expression levels of the identified proteins showed a strong correlation between experiments (Spearman coefficient of 0.9774). (D-E) The proteomic data was further analysed to look for differences between the uninfected BMDMs and those infected with *C. albicans*. For each experiment a subset of proteins were regarded as being regulated by *C. albicans* infection if the showed a log2 fold change more that 1 standard deviation away from the median fold change and a q < 0.05 (see volcano plots in the main figure 1); where up-regulated proteins were present in ≥3 of the infected replicates, and down-regulated proteins were present in ≥3 unstimulated replicates. For proteins as regulated in experiment 1, the log2 fold change in experiment 1 plotted against the log2 fold change in experiment 2 is shown in (D). The -log10 q values for experiment 2 are given by the colour gradient. (E) shows the equivalent analysis for proteins identified as regulated in experiment 2.

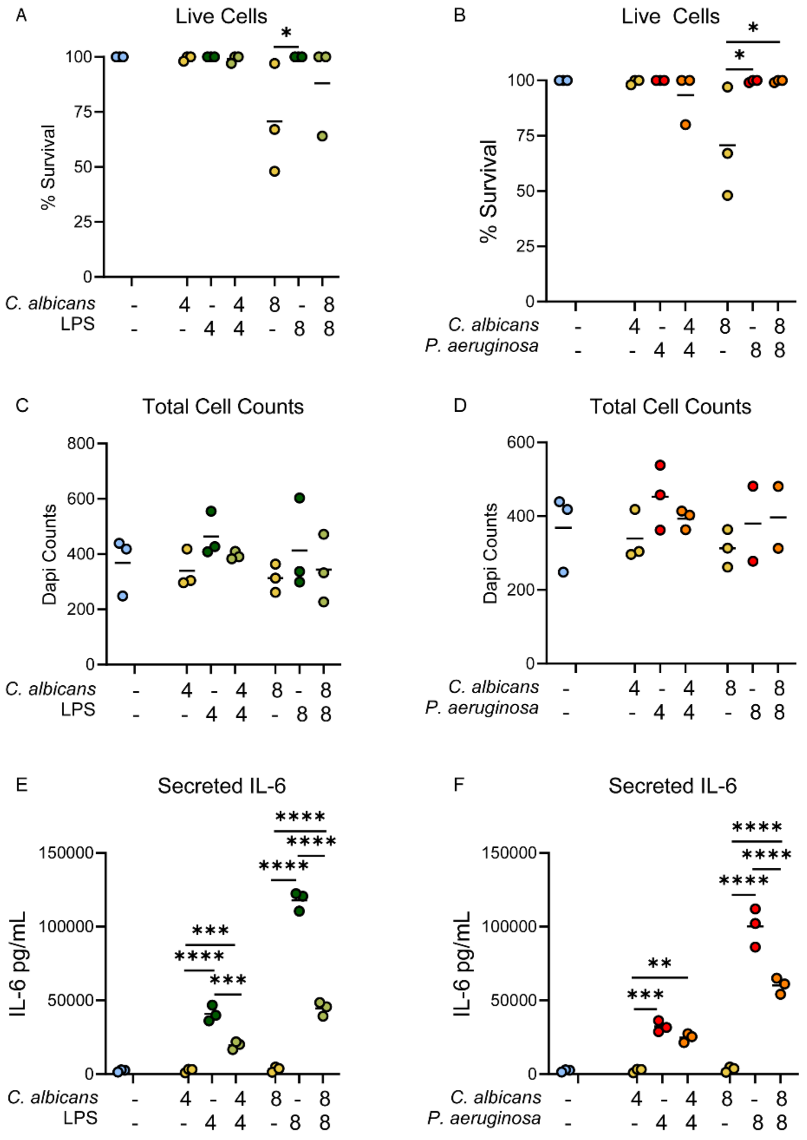

Supplementary Figure 3 Repression of IL-6 production in BMDMs by *C. albicans* did not correlate to changes in macrophage survival.

BMDMS were left unstimulated or treated with the indicated combinations of 100ng/ml LPS, *C. albicans* infection (MOI 4:1) or *P. aeruginosa* infection (MOI 10:1). After 4 or 8 h, BMDM survival and IL-6 levels in the media were determined as described in the methods. (A, B) The percentage of live macrophages based on exclusion of the DNA binding dye Sytox green dye. (C,D) Cells were imaged and the number of macrophages per field of view determined. (E, F) IL-6 levels present in the cell culture media. Data shows results from 3 biological replicates. Data was analysed by two way ANOVA (C-G) or, to correct for unequal variance, two way ANOVA on log10 transformed data (A, B,). F and p values for the ANOVA are shown in supplemental table 8. An adjusted *p*-value of < 0.05 is indicated by *, < 0.01 by **, <0.001 by *** and < 0.0001 by ****.

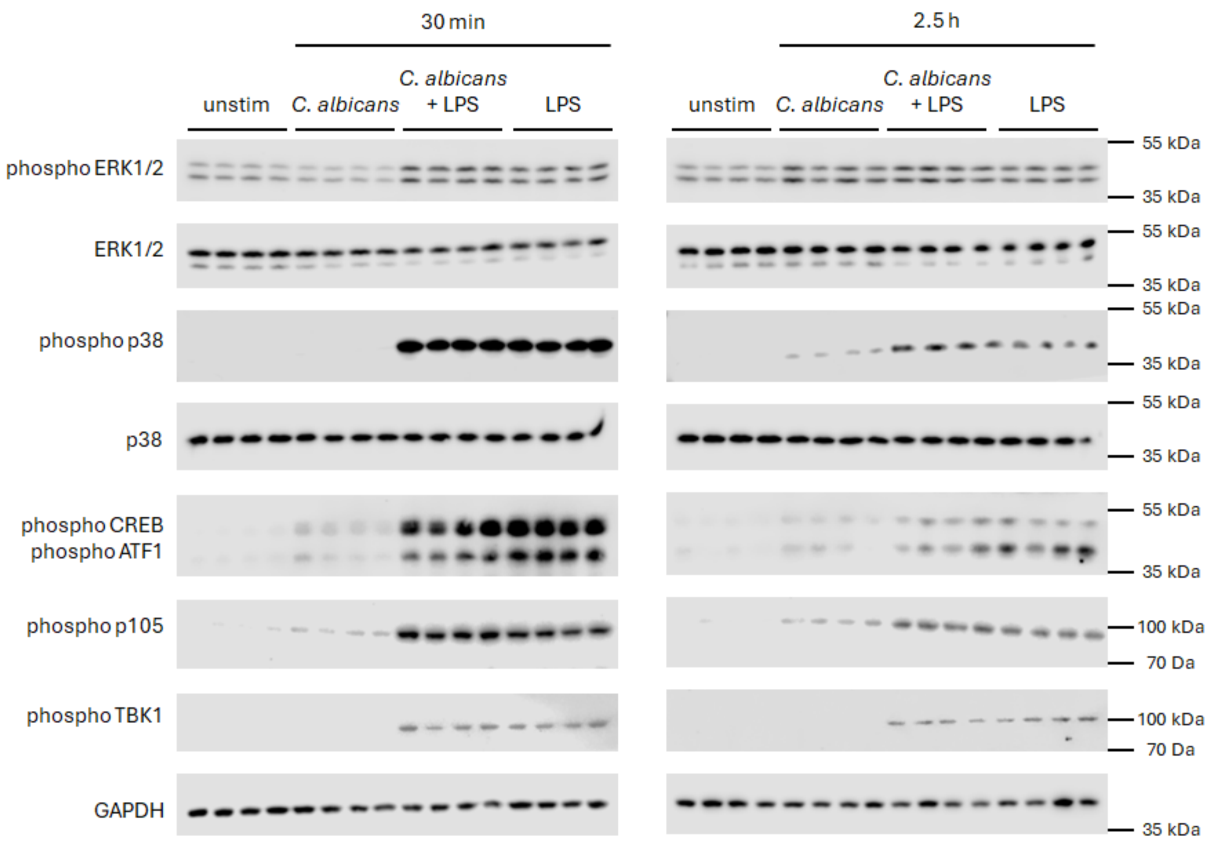

Supplementary Figure 4 ***C. albicans* minimally activates MAPK and NFκB signalling in comparison to LPS.**
BMDMs were left unstimulated (unstim) or infected with live *C. albicans* (MOI 4), 100 ng/mL LPS or co-treated with a combination of both *C. albicans* and LPS for either 30-minutes or 2.5-hours and lysed for immunoblot analysis. Levels of the indicated proteins are shown. Full membrane scans are shown in supplemental figure 11.

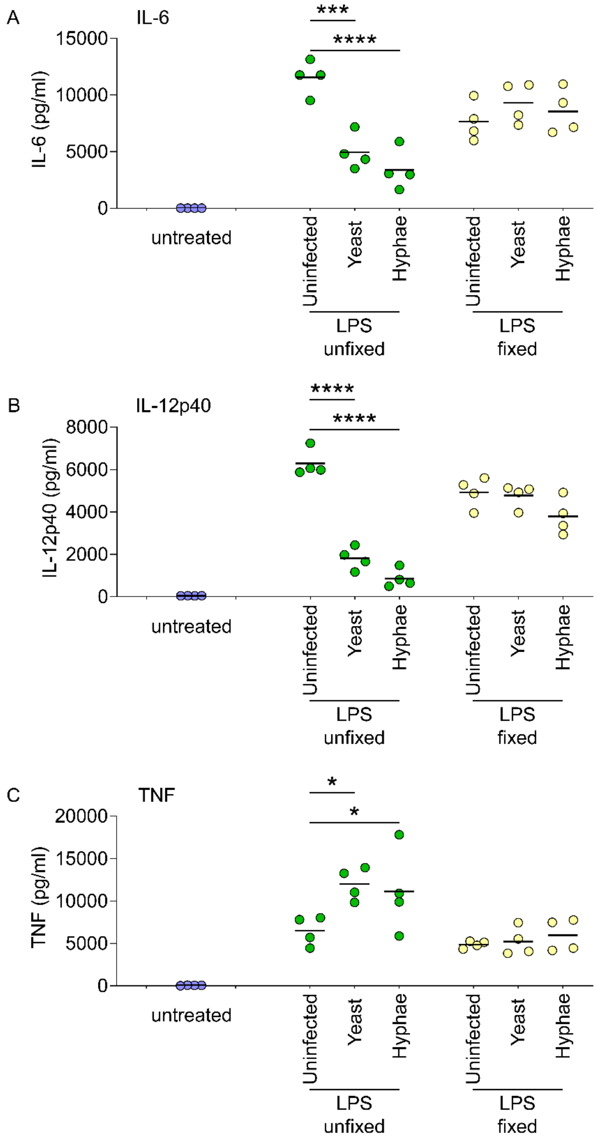

Supplementary Figure 5   Fixation of *C. albicans* yeast or hyphae reduces their ability to repress LPS induced IL-6 production.

BMDMs were left uninfected or stimulated LPS (100 ng/ml) for 8h in the presence or absence of the indicated forms of *C. albicans*. Levels of IL-6, IL-12p40 and TNF in the media were then determined. For these experiments, C. *albicans* was grown as a yeast in YPD media; after washing, this was either used directly in macrophage infection experiments (live yeast) or allowed to transition into a hyphal morphology by culture in DMEM for 2-hours being used (live hyphae). Alternatively, aliquots of both yeast and hyphal *C. albicans* were inactivated by fixation in paraformaldehyde as described in the methods (fixed yeast / fixed hyphae). To control for any potential carry over of paraformaldehyde, an equivalent volume of supernatant from the final fixed *C. albicans* suspension was also added to macrophages in combination with LPS. Data was analysed by 2 way ANOVA with Tukey's post hoc testing with comparisons to the uninfected LPS condition shown ($p < 0.05$ indicated by *, $p < 0.01$ by **, p< < 0.001 by *** and $p < 0.0001$ by ****). F and p values for the ANOVA are (A) Infection F=9.22, p=0.0018, fixation F=7.02, p=0.0163, interaction F=16.72, p<0.0001; (B) Infection F=56.32, p<0.0001, fixation F=33.72, p<0.0001, interaction F=30.55, p<0.0001; (C) Infection F=3.54, p=0.0505, fixation F=19.45, p=0.0003, interaction F=2.22, p=0.1378.

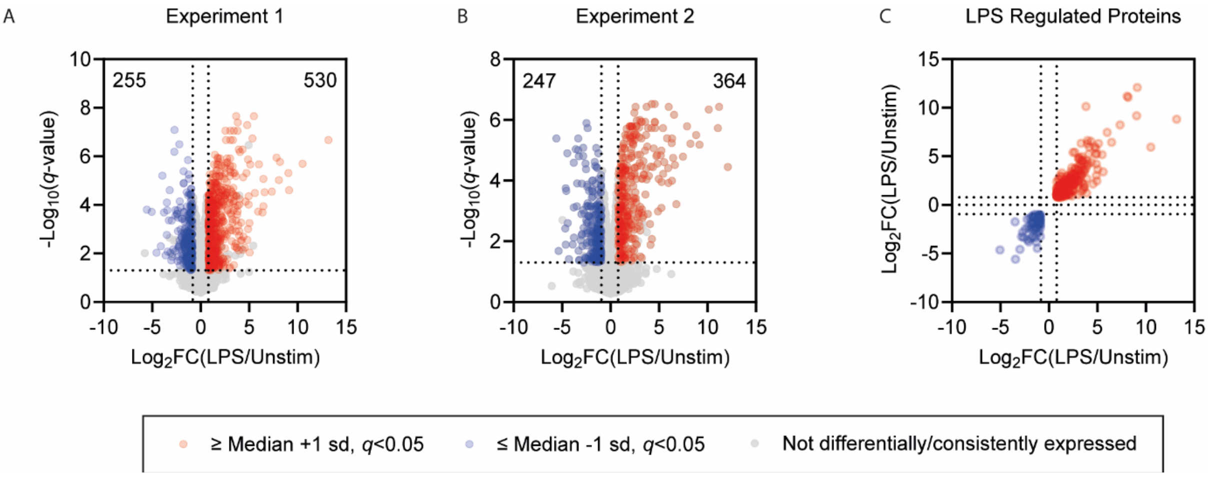

Supplementary Figure 6 Identification of LPS regulated proteins in BMDMs.
BMDMs were either left unstimulated or co-infected with *C. albicans* and LPS or LPS alone for 8-hours and then lysed for proteomic analysis. Two separate experiments, each with 4 biological replicates were performed. For a comparison of unstimulated BMDMs and LPS stimulation in each experiment (A, B), up-regulated proteins were defined as having a log2 fold change (FC) of more than 1 standard deviation above the median, and a *q*-value < 0.05 and being present in 3 or more of the LPS stimulated replicates. Down regulated proteins were defined as having a log2 fold change (FC) of more than 1 standard deviation below the median, and a *q*-value < 0.05 and being present in 3 or more of the unstimulated samples. Additionally, proteins present in all of the LPS but none of the unstimulated replicates were regarded as up regulated while proteins present in all of the unstimulated replicates but none of the down regulated ones were regarded as down regulated. These proteins would not have generated a q value, but added 129 and 87 upregulated proteins to the upregulated list for experiment 1 and 2 respectively and 27 and 60 proteins to the down-regulated list. A refined list of proteins showing consistent regulation by LPS was generated by taking those proteins that pass the above cut-offs in both experiments. An xy plot for the log2 FC values in the two experiments is shown in (C), and these values had a Spearman corelation co-efficient of 0.8709. The refined list of LPS regulated proteins is given in Supplementary Table 3 and was used for the analysis in Figure 5.

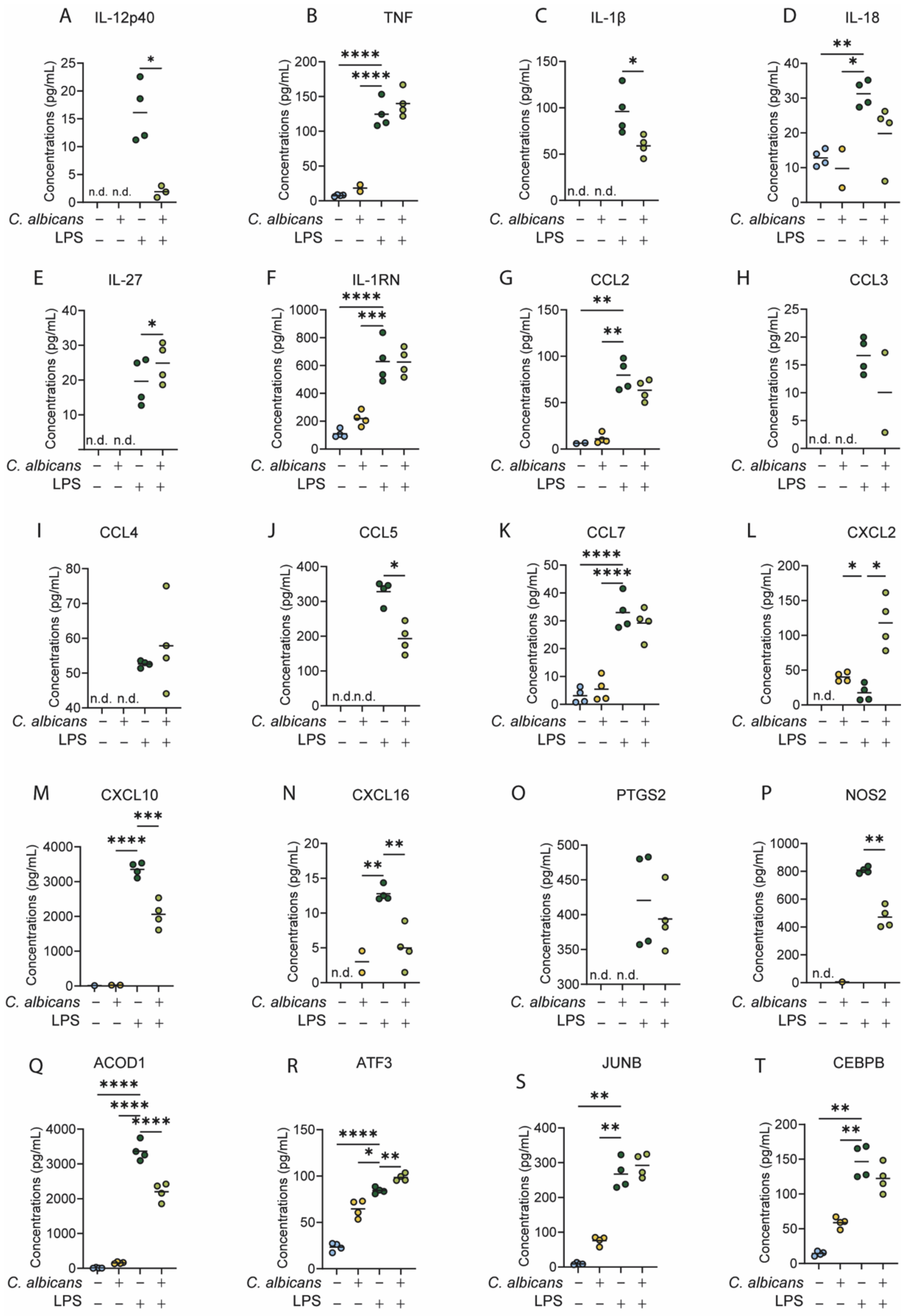

Supplementary Figure 7 Effect of *C. albicans* on the induction of selected LPS regulated proteins in BMBMs.

(A-T) Protein concentrations from the indicated proteins from proteomic Experiment 1 (equivalent data for experiment 2 is given in Figure 6 in the main paper). Statistics are summarised in Supplementary Table 4, where all post-hoc comparisons were made to LPS. The mean of each condition group is displayed as a black line. Adjusted *p*-values≤ 0.05 (*), adj. p ≤ 0.01 (**), adj. p≤0.001 (***), adj. p≤0.0001 (****).

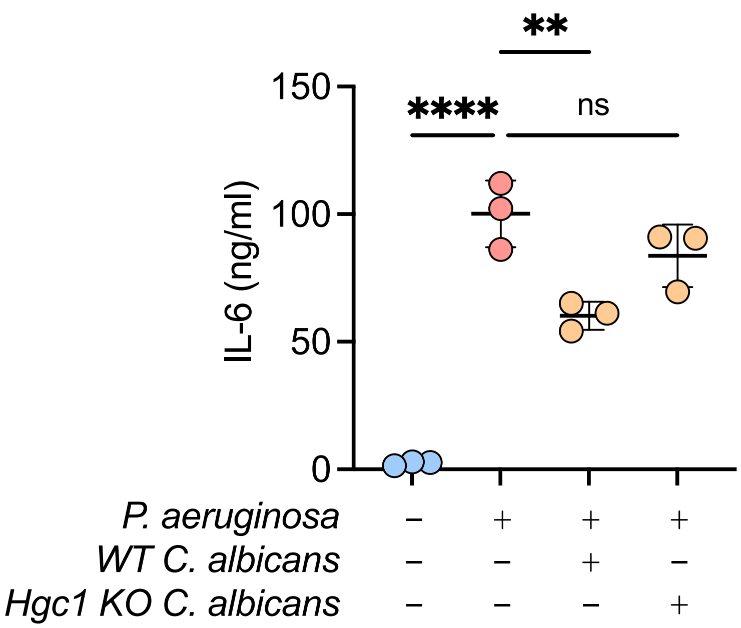

Supplementary Figure 8 Identification of *P. aeruginosa* regulated proteins in BMDMs.
BMDMs were left uninfected or infected with *P. aeruginosa* (MOI of 10) in the presence or absence of wild type (WT) *C. albicans* or Hgc1 knockout (KO) C. albicans (MOI of 4)or a combination of both micro-organisms for 8-hours. Media was then collected and analysed of the level of IL-6. Symbols show data from macrophages from separate mice, with mean values shown by a vertical line. Data was analysed by one-way ANOVA ($F = 63.53$, $p < 0.0001$) with post hoc testing using Dunnett's multiple-le comparison test. For compassions to the only condition, $p < 0.01$ is indicated by ** and $p < 0.0001$ by ****. ns indicates $p > 0.05$.

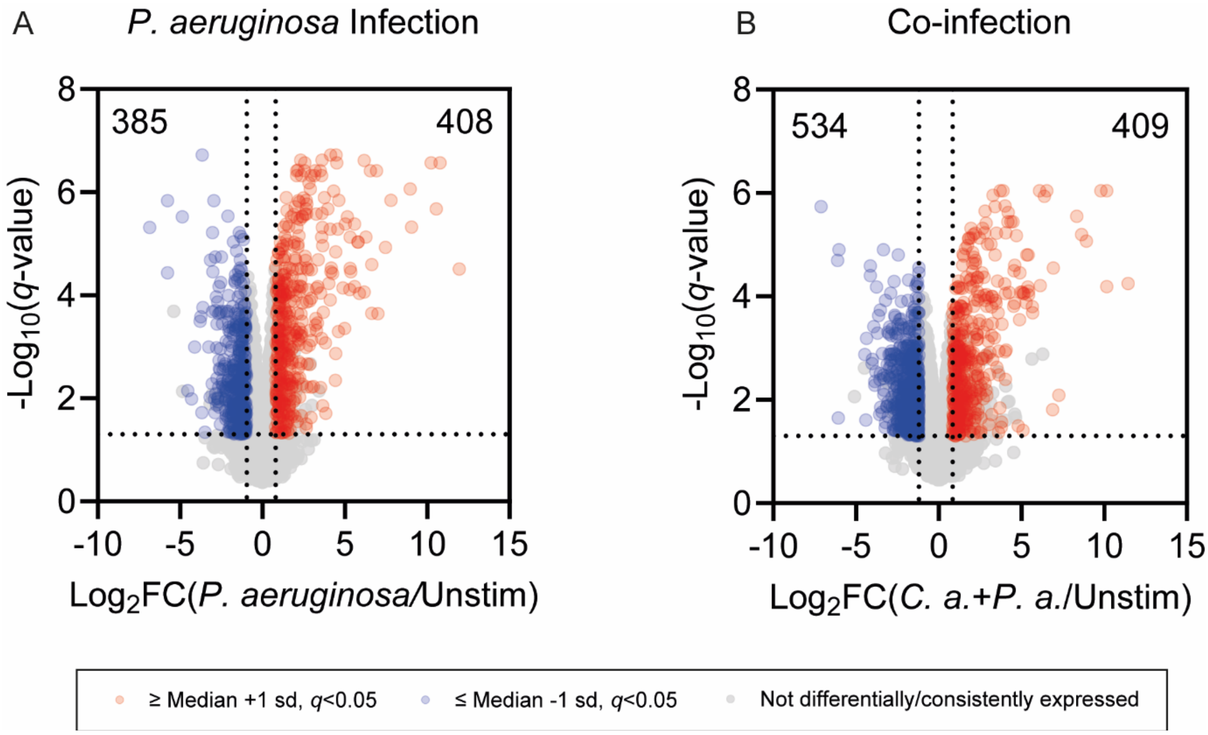

Supplementary Figure 9 Identification of *P. aeruginosa* regulated proteins in BMDMs.
BMDMs were left unstimulated or co-infected with *C. albicans*, *P. aeruginosa* or a combination of both micro-organisms for 8-hours and then lysed for proteomic analysis. For a comparison of uninfected and *P. aeruginosa* infected BMDMs (A), up-regulated proteins were defined as having a log2 fold change (FC) of more than 1 standard deviation above the median, and a *q*-value < 0.05 and being present in 3 or more of the *P. aeruginosa* infected replicates. Down regulated proteins were defined as having a log2 fold change (FC) of more than 1 standard deviation below the median, and a *q*-value < 0.05 and being present in 3 or more of the uninfected samples. Additionally, proteins present in all of the LPS but none of the uninfected replicates were regarded as up regulated while proteins present in all of the unstimulated replicates but none of the down regulated ones were regarded as down regulated. These proteins would not have generated a q value, but added 79 upregulated 47 downregulated proteins to the list and was used for the analysis in Figure 7 and shown in Supplementary Table 5. A volcano showing changes between uninfected and co-infected BMDMs is shown for comparison (B).

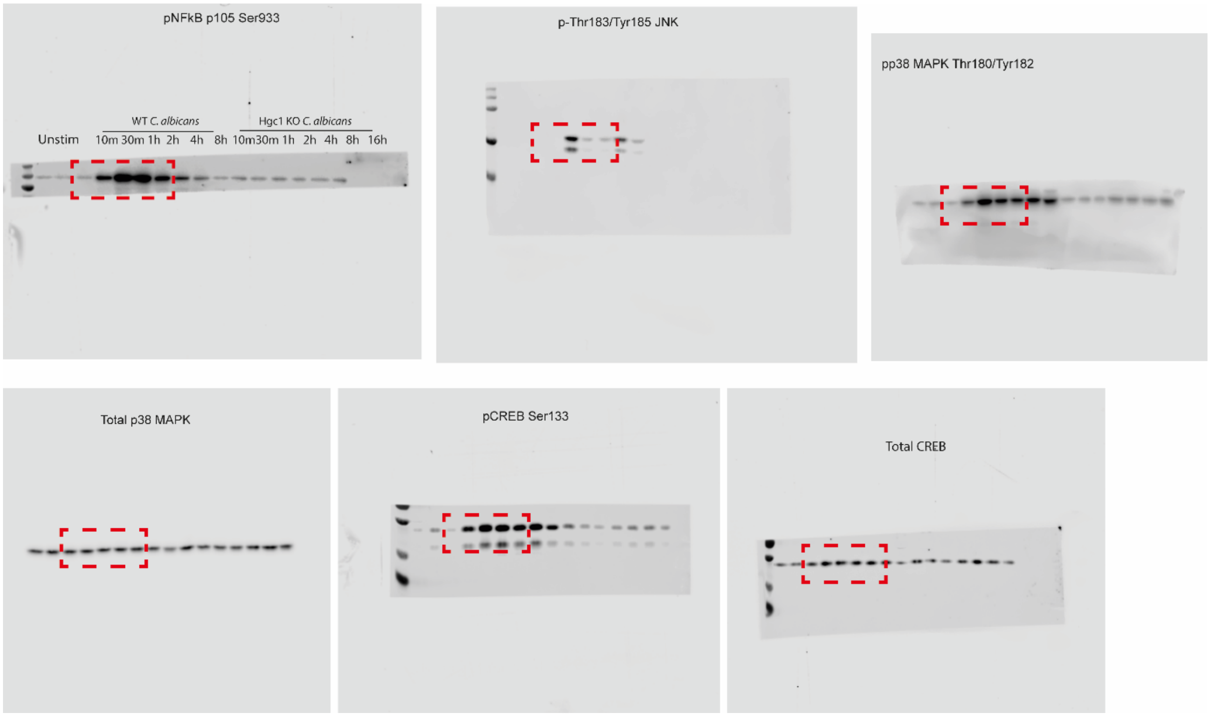

Supplementary Figure 10 Immunoblots of Supplementary Figure 1.
Membranes from western blot in Supplementary Figure 1, where proteins are noted on membranes. Red boxed indicated the data used in supplementary figure 1.

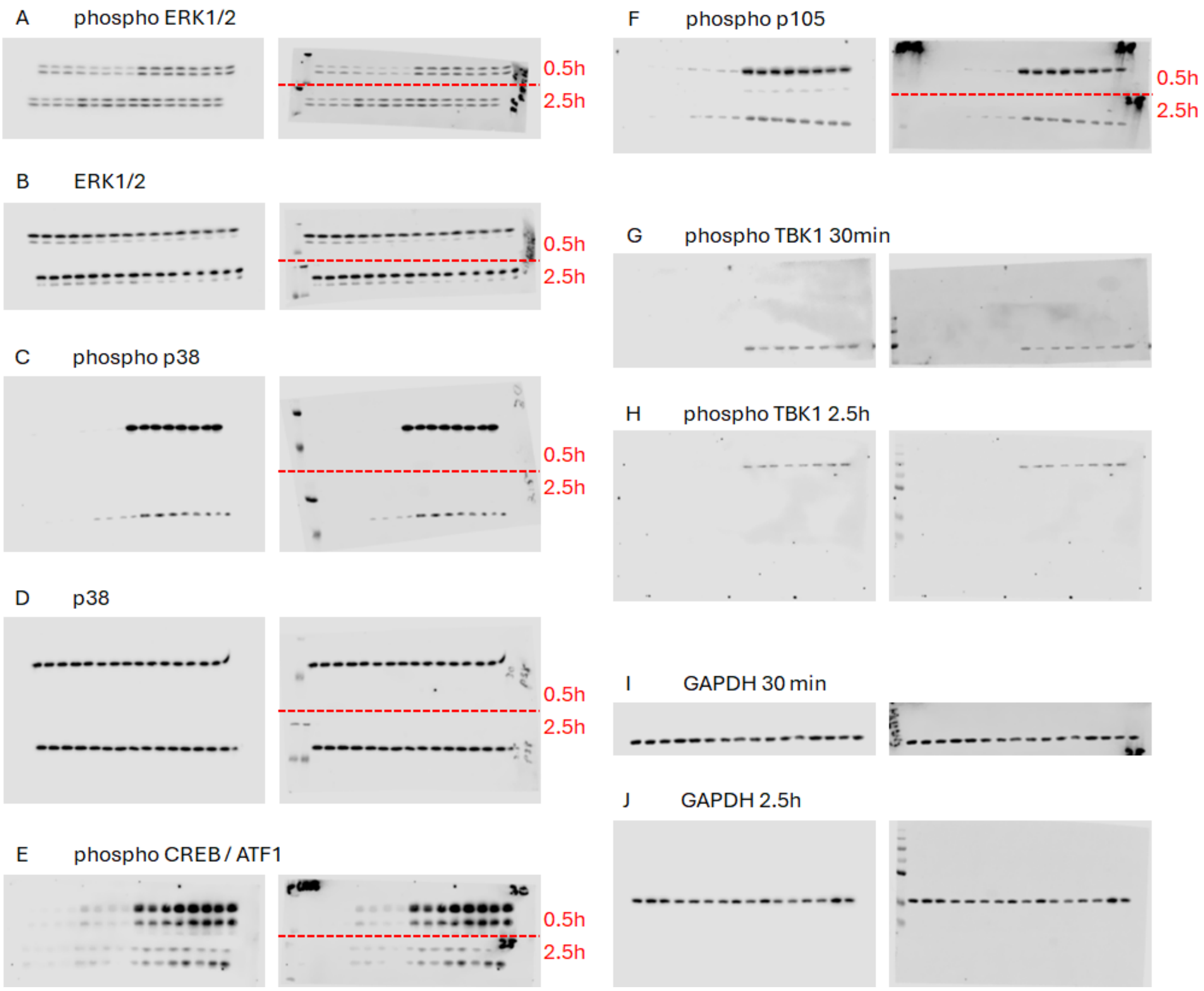

Supplementary Figure 11. Scans of immunoblots for Supplementary Figure 4.
Lysates to examine signalling in response to C. albicans infection and/or LPS stimulation were generated at 30min and 2.5h time points. Samples were analysed for the levels of (A) phospho ERK1/2, (B) total ERK1/2, (C) phospho p38, (D) total p38, (E) phospho CREB/ATF1, (F) phospho p105, (G, H) phospho TBK1 and (I, J) total GAPDH. For A to F, the 30min and 2.5h time points were run on separate gels. The section of the gel corresponding to the molecular weight of the protein to be analysed was the excised and the slices for the 30min and 2.5h time points transferred on to the same nitrocellulose membrane for blotting. The division between the 30min and 2.5h time point is indicated by the red dotted line. For GAPDH and phopho TBK1 separate membranes were analysed for the two time points. Membranes were imaged on a Licor Odyssey XF system with collection in the chemiluminescent channel (to detect the HP-conjugated secondary antibody and fluorescent channels to detect the pre-stained molecular weight markers. For each membrane images of the chemiluminescent channel only are shown on the left and were used to prepare supplementary figure 4. Images the same exposure of the chemiluminescent channel overlaid with the fluorescent channels are shown on the right to allow visualisation of the markers.
